# Evaluating the Durability of Perfluorosulfonic Acid Membranes in Fuel Cells Using Combined Open-Circuit Voltage-Accelerated Stability Testing

**DOI:** 10.3390/polym16101348

**Published:** 2024-05-09

**Authors:** Juniko Nur Pratama, Hyunwoo Song, Hansung Kim, Hyejin Lee, Dongwon Shin, Byungchan Bae

**Affiliations:** 1 Fuel Cell Laboratory, Korea Institute of Energy Research, Daejeon 34129, Republic of Korea; junikotama@kier.re.kr (J.N.P.); hyunwoo0225@kier.re.kr (H.S.); dwshin@kier.re.kr (D.S.); 2 Renewable Energy Engineering, University of Science and Technology, Daejeon 34113, Republic of Korea; 3 Department of Chemical and Biomolecular Engineering, Yonsei University, Seoul 03722, Republic of Korea; elchem@yonsei.ac.kr; 4 Hydrogen Energy Engineering, University of Science and Technology, Daejeon 34113, Republic of Korea

**Keywords:** proton exchange membrane fuel cells, reinforced membrane, combined open-circuit voltage, accelerated stress test

## Abstract

This study evaluates the chemical and mechanical durability of membranes used in proton exchange membrane fuel cells, highlighting the essential role of electrochemical tests in understanding the relationship between durability and performance. Our methodology integrates various electrochemical evaluation techniques to assess the degradation of perfluorosulfonic acid (PFSA) membranes. The results highlight the considerable improvement in the chemical and mechanical durability of annealed 3M PFSA-reinforced composite membranes (RCMs) compared with their non-annealed counterparts and other membrane types, indicating their superior resilience under challenging conditions. Moreover, the results of using a combined open-circuit voltage-accelerated stability testing protocol demonstrate that annealed 3M PFSA RCMs exhibit enhanced resilience, reaching 18,000 cycles before failure, considerably outperforming NR 211 (5000 cycles) and other membranes. In addition, membrane deterioration over time can be precisely measured by interpreting electrochemical indicators (electrochemically active surface area, circuit resistance, high-frequency resistance, and proton resistance). This approach provides a clear relationship between electrochemical data and durability, offering a comprehensive understanding of how different membranes withstand operational stresses.

## 1. Introduction

Fuel cells are important devices that allow the direct transformation of chemical energy from fuels into electrical power. These devices are the result of developments in electrochemical engineering. In particular, proton exchange membrane fuel cells (PEMFCs) are noted for their high power efficiency and minimal environmental impact. The key component of PEMFCs is the perfluorosulfonic acid (PFSA) ionomer membrane, which exhibits excellent proton conductivity and robust physicochemical properties. This membrane features a super hydrophobic polytetrafluoroethylene (PTFE) backbone that increases the cell’s structural strength through its crystalline architecture and hydrophilic side chains, which contain sulfonic acid groups that facilitate proton transfer and create essential ion transport channels. Nafion^®^ membranes are popular, commercially available PFSA-based membranes from DuPont, which have been used since the early stages of PEMFC studies because of their good proton conductivity and gas separation performance [1,2,3].

In automotive applications, PEMFCs are subjected to challenging operating conditions characterized by repetitive hydration and dehydration cycles, known as wet–dry cycling. This process exerts considerable mechanical stress on membranes, potentially leading to their failure, owing to tearing or breakdown, which adversely affects fuel cell performance [4]. Moreover, the catalytic reaction between hydrogen and oxygen generates hydrogen peroxide or radicals within the membrane electrode assembly (MEA). These reactive species pose a considerable risk to the structural integrity of PFSA membranes because they can break down the polymer backbone and side chains, leading to oxidative degradation. This degradation accelerates a decrease in the molecular weight of membranes and compromises the durability of fuel cells. Thus, it is important to address the challenges posed by wet–dry cycling and radical-induced chemical degradation to improve the longevity and reliability of PEMFC systems and ensure their sustainable operation under challenging conditions in automotive applications [5].

To counteract the harmful effects of wet–dry cycling and oxidative degradation, several innovative solutions, such as incorporating radical scavengers [6,7,8,9] and reinforcing PFSA membranes with porous PTFE matrices [10,11], have been investigated. These solutions are accurately assessed using accelerated stability testing (AST), which is a pivotal protocol for evaluating membrane durability to enhance fuel cell lifetimes under operational stresses. By mimicking real operating conditions, i.e., temperature, humidity, and open-circuit voltage (OCV), to which a fuel cell is often subjected, AST provides a critical framework for assessing the effectiveness of these technological enhancements [12,13]. Owing to the importance of standardized testing protocols, the United States (US) Department of Energy (DOE) and the New Energy and Industrial Technology Development Organization (NEDO, Japan) have established comprehensive AST guidelines [5,14,15,16]. These protocols are widely adopted across the industry, and they involve separate tests for evaluating chemical and mechanical degradations based on OCV holding and wet–dry cycling tests, respectively.

However, a combined AST method, integrating the OCV and wet–dry cycling tests under modified conditions, has been developed to reduce the overall test duration and accelerate the evaluation process while accurately predicting membrane durability. Specifically, in the DOE combined OCV (COCV) protocol, membrane durability assessment is conducted under conditions of 90 °C and 100% relative humidity (RH), incorporating a wet–dry cycle for 30/45 s while supplying hydrogen and oxygen to the anode and cathode sides, respectively. This protocol can efficiently reduce the test duration; however, it cannot separately examine each degradation mechanism because it simultaneously evaluates chemical and mechanical durability. Despite this limitation, COCV is a reliable protocol for rapidly assessing the durability of PFSA membranes under operating conditions [15].

Despite the broad application of COCV-AST, few studies have explored the diverse electrochemical data from electrochemical evaluations and the behavior of membranes under AST conditions. Thus, detailed investigations into the impacts of COCV-AST on membrane performance and longevity will fill a large gap in the literature. To this end, we conduct a comprehensive analysis of the COCV-AST methodology. The present study elucidates the electrochemical behavior and degradation mechanisms of PFSA membranes during COCV-AST by investigating complex changes within the membrane throughout the testing process. This endeavor is critical for advancing the field, offering insights for improving diagnostic technologies with the aim of enhancing membrane durability [16].

Our previous study focused on the thermal behavior of PFSA-reinforced composite membranes (RCMs) and their responses to COCV-AST conditions, primarily comparing the performance and durability of reinforced and non-reinforced membranes [10]. We obtained valuable insights regarding the durability and operational resilience of PFSA RCMs under accelerated stress. COCV-AST conditions, which subject membranes to wet and dry cycles at high temperatures, are crucial in assessing the performance of membranes because they simulate the real-world operating conditions of PEMFCs. By exposing these membranes to extreme conditions, researchers can evaluate membrane durability and operational resilience, and obtain key insights into their potential for long-term usage in fuel cell applications.

However, electrochemical dynamics, including cyclic voltammograms (CVs), linear sweep voltammograms (LSVs), and electrochemical impedance spectroscopy (EIS) data, were not explored extensively. Therefore, this study extends our previous work by offering a comprehensive electrochemical analysis of membranes using CVs, LSVs, and EIS data. In addition, it provides a more nuanced understanding of membrane behavior and degradation mechanisms under accelerated stress conditions, which will contribute to advancing diagnostic technologies and improving membrane durability [16].

Furthermore, we investigate the electrochemical characteristics of Nafion^®^ and Nafion^®^ HP as the benchmark for non-reinforced membrane and RCM, respectively, and compare them with a custom-made RCM using 3M ionomers. Nafion^®^ NR 211 is a commercially available PFSA membrane widely used in PEMFC applications requiring homogeneous membranes [2]. Meanwhile, Nafion^®^ HP is a reinforced membrane used in applications such as fuel cells, electrolysis, and other electrochemical processes [17]. We reveal the complex electrochemical behaviors of these membranes and their corresponding MEAs by periodically and systematically obtaining their CV, LSV, and EIS data throughout COCV-AST.

## 2. Materials and Methods

### 2.1. Materials and Chemicals

Laminated Nafion^®^ NR 211 and Nafion^®^ HP were obtained from the Chemours Company FC, LLC, Wilmington, NC, USA and Ion Power, Inc., New Castle, UK, respectively. 3M PFSA powder ionomers (equivalent weight = 729, E-21669D) were purchased from 3M Company, Minnesota, MN, USA. *N*-methyl-2-pyrrolidone (NMP, anhydrous, 99%, CAS 872-50-4), which was used as the solvent to dissolve the 3M powder ionomers, was purchased from Sigma-Aldrich, Missouri, TX, USA. The porous polytetrafluoroethylene PTFE substrate was obtained from Donaldson Company, Minneapolis, MN, USA.

### 2.2. Fabrication and Annealing of Perfluorosulfonic Acid (PFSA) Membranes

To prepare a 3M 729 solution (10 wt%), 3M perfluorosulfonic acid (PFSA) powder was dissolved in *N*-methyl-2-pyrrolidone (NMP) solvent at 25 °C by stirring with a magnetic stirrer bar overnight at a moderate mixing speed. Subsequently, the solution was filtered using a 0.45 µm polytetrafluoroethylene (PTFE) syringe filter from Whatman, Buckinghamsire, UK. The filtered solution was then readied for casting and could be combined with a porous PTFE substrate to facilitate the fabrication of reinforced composite membranes (RCMs).

**Figure 1:**
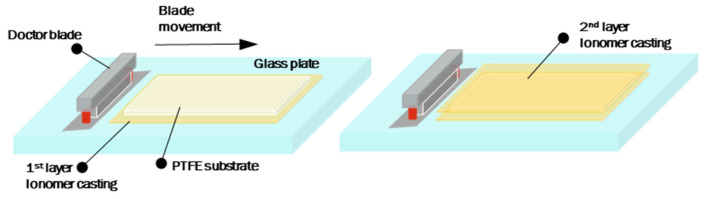
Fabrication process of three-layer reinforced membranes using the doctor blade method.

Figure 1 illustrates the fabrication process of a three-layer RCM, which involved the following steps: (i) the casting of the PFSA solution onto a glass plate using the doctor blade, (ii) placing a porous PTFE substrate over the lower PFSA layer, (iii) forming an upper PFSA layer by casting a second PFSA solution using the doctor blade, and (iv) allowing the layered membranes to dry overnight on a hot plate at 70 °C. Subsequently, RCMs derived from 3M PFSA were labeled as 3M PFSA RCMs. They have a targeted thickness of approximately 25 µm to facilitate a comparison between the Nafion^®^ NR 211 and HP membranes. The membrane thickness was adjusted by controlling the height of the doctor blade. For drying, RCMs were transferred from the casting glass plate to a vacuum oven at 80 °C for 2 days.

Moreover, the 3M PFSA RCMs and Nafion HP membranes were treated with 3M HCl at 80 °C for 6 h. This treatment eliminated the residual NMP solvent while protonating membranes. Next, the membranes were thoroughly cleaned, rinsed using distilled water, and air-dried overnight at 25 °C. Finally, 3M PFSA RCMs were annealed at 200 °C for 1 h in a vacuum oven. Meanwhile, the Nafion HP membrane underwent the same annealing process in a vacuum oven under identical conditions for 1 h but at a lower temperature (170 °C).

### 2.3. Combined OCV Wet–Dry Cycling Test

Four membranes were prepared for the MEA (area ≈ 9 cm^2^) fabrication using the decal transfer method employing carbon-supported platinum (TEC10F50E, Tanaka Kikinzoku Kogyo K.K., Tokyo, Japan) [8]. The MEA was obtained at a platinum loading of 0.3–0.35 mgPt cm^−2^. An Aquivion ionomer (D83-24B, Solvay, Brussels, Belgium) was used as the ionomer binder for the electrode layer.

COCV-AST was conducted using a protocol validated by the US DOE. The COCV-AST procedure involved subjecting the MEA to conditions of 90 °C with simultaneous wetting and drying cycles at an RH of 0–100%. An RH of 100% was confirmed using an in situ temperature and humidity sensor (PTU300 Combined Pressure, Humidity, and Temperature Transmitter, Vaisala, Helsinki, Finland). Hydrogen gas was introduced to the anode at a flow rate of 6.67 × 10^−6^ m^3^ s^−1^ (400 sccm), and high-purity air was supplied to the cathode at a flow rate of 6.67 × 10^−6^ m^3^ s^−1^ (400 sccm). OCV was continually monitored using a potentiostat (ZIVE SP2, WonATech, Seoul, Republic of Korea).

In a single cycle (denoted 30/45 s) within the wet–dry cycle test, an MEA was subjected to 30 s of dry conditions (RH = 0%) followed by 45 s of wet conditions (RH = 100%). Additionally, at 90 °C and 100% RH, LSV, CV, and high-frequency resistance (HFR) measurements were performed using a potentiostat (HCP-803, Bio-Logic Science Instruments, Grenoble, France) every 1000 cycles until the membrane and MEA fail. Moreover, before and after the interim analysis, LSV, CV, and HFR were measured at 80 °C and 100% RH. Hydrogen gas was fed to the anode at a flow rate of 3.33 × 10^−6^ m^3^ s^−1^ (200 sccm), and nitrogen gas was fed to the cathode side at 1.67 × 10^−5^ m^3^ s^−1^ (1000 sccm) for LSV and HFR. In contrast, only hydrogen gas was fed to the anode side at a flow rate of 3.33 × 10^−6^ m^3^ s^−1^ (200 sccm) for CV measurements. Cycling was terminated when the crossover current density exceeded ten times the initial value or when OCV decreased to 80% of its initial value, whichever occurred first.

The crossover current density was determined from the intersection of the *y*-axis with an extrapolated line drawn between 0.4 and 0.5 V from the LSV analysis data. The reciprocal of the slope of this line indicates short-circuit resistance [18].

The CV measurements were used to calculate the electrochemically active surface area (ECSA, in m^2^/g) of the catalyst as follows [19]:

(1) $$ECSA=\frac{QH}{m\times qH}$$

where QH is the charge density (mC/cm^2^) obtained by integrating the area of the hydrogen desorption peak below 0.4 V after applying the double-layer correction, qH is the charge associated with monolayer hydrogen adsorption on the platinum surface (assumed to be 0.21 mC/cm^2^), and m indicates the platinum loading (mg/cm^2^).

EIS was conducted to measure HFR and proton resistance over a scanning frequency range of 1 Hz–20 kHz. The working electrode potential (EWE) was set to 0.404 V versus the reference, and measurements were taken at intervals of 1 mA/s. HFR was determined by extrapolating the high-frequency end of the Nyquist plot and obtaining the intercept on the real axis. The 45° segment of the Nyquist plot predicted by the transmission-line model provided information necessary to calculate electrode proton resistance [20].

The COCV-AST, LSV, CV, and HFR measurements were performed simultaneously until the membrane inside the electrode assembly failed. Each MEA was constructed using the same decal transfer method simultaneously and under the same conditions to minimize the possibility of anomalous results.

## 3. Results and Discussion

**Figure 2:**
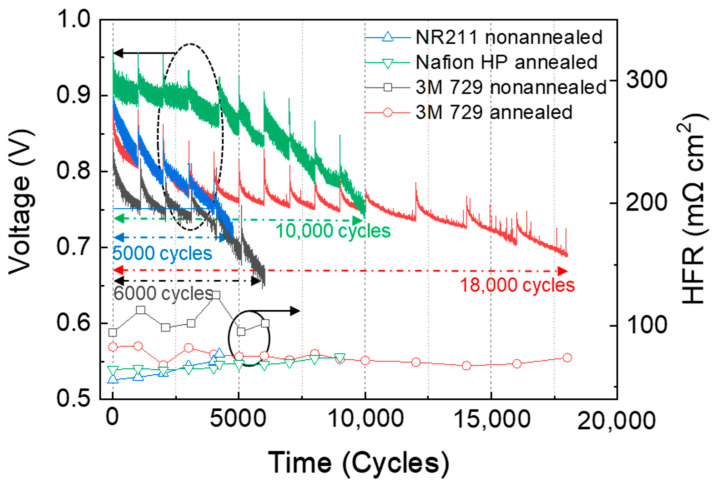
OCV and HFR changes in single cells assembled using NR 211, annealed Nafion HP, and non-annealed and annealed 3M PFSA RCM (determined based on chemical and mechanical cycle tests at 90 °C over a cycle duration of 30/45 s).

The measured parameters of the NR 211 membrane were used as the reference values to compare with those of non-annealed 3M PFSA RCM. Furthermore, two annealed RCMs were compared, namely Nafion HP and 3M PFSA RCM. To ensure consistency across tests, all four membranes were prepared with a uniform thickness of 25 µm and were incorporated into MEAs containing identical catalyst layers. The evaluation of these MEAs during COCV-AST involved monitoring voltage and HFR over the test duration (Figure 2).

Throughout the COCV-AST study, the two pivotal parameters were closely monitored: OCV and HFR. OCV reflects the equilibrium voltage of the fuel cell in the absence of an external load, membrane gas permeability, and the activity of the catalyst layer. Exposure to OCV induces the generation of oxidative species such as peroxides and radicals at both electrodes, which causes the deterioration of the binder ionomer of the electrode and membrane [19,21]. The formation of these oxidative species at the cathode and anode is given below:

At the cathode:

(2) $$H_{2}+O_{2}\to H_{2}O_{2}\;\mathrm{(chemically formed)}$$

At the anode:

(3) $$H_{2}+O_{2}\to H_{2}O_{2}\;\mathrm{(chemically formed)}$$

(4) $$2H^{+}+O_{2}+2e^{-}\to H_{2}O_{2}\;\mathrm{(electrochemically formed)}$$

(5) $$\frac{1}{2}H_{2}O_{2}\to {HO}_{2}^{•}\;\mathrm{(chemically formed)}$$

Thus, maintaining a stable OCV is a practical criterion for assessing the oxidative durability of membranes [5]. Meanwhile, HFR measures MEA ohmic resistance, which is predominantly governed by ohmic resistance within membranes and the catalyst layer. HFR tends to increase because of the oxidative degradation of the membrane, reduction in the number of ion exchange sites, and degradation of the binder in the catalyst layer. This membrane degradation is more pronounced during COCV-AST than during OCV tests. The repeated cycles of swelling and drying inherent to COCV testing exert considerable physical stress on the membrane, ultimately causing the formation of pinholes and subsequent tearing [22,23,24]. This progression reflects the harsh environmental impact of these testing protocols on membrane resistance [4,5].

Figure 2 shows the durability outcomes obtained from COCV-AST tests, where NR 211 and non-annealed 3M PFSA RCM failed after 5000 and 6000 cycles, respectively. In contrast, annealed Nafion HP and 3M PFSA RCM exhibited a more gradual voltage decline, ultimately failing after a considerably higher number of cycles, i.e., 10,000 and 18,000 cycles, respectively. The *y*-axis on the left side in Figure 2 corresponds to the durability outcomes of different membranes under COCV-AST conditions, which provided valuable insights into their long-term performance and durability under accelerated stress conditions. The *y*-axis on the right side indicates the different HFR patterns of the investigated membranes, which also reflected their performance. Specifically, the HFR of NR 211, Nafion HP, and non-annealed 3M PFSA RCM increased as COCV-AST progressed, reflecting their deterioration. In contrast, annealed 3M PFSA RCM managed to maintain a relatively stable HFR, indicating its superior endurance and enhanced chemical and mechanical durability.

Comparing the performance of NR 211 and Nafion HP showed that the lifetime of Nafion HP nearly doubled, which could be attributed to the reinforcing impact of the PTFE matrix on the chemical and mechanical durability of Nafion HP, coupled with the annealing process. The incorporation of PTFE enhanced the mechanical properties of the NR 211 membrane, and the annealing process facilitated the reorganization of polymer chains. This reorganization reduced the free volume, increased the crystallinity, and enhanced the chemical and mechanical durability of the membrane. This synergistic effect effectively mitigated internal stress in the membrane, reducing the incidence of microcracks and pinhole formation during COCV-AST and prolonging the operational lifespan of the NR 211 membrane [5,10,15,23].

**Figure 3:**
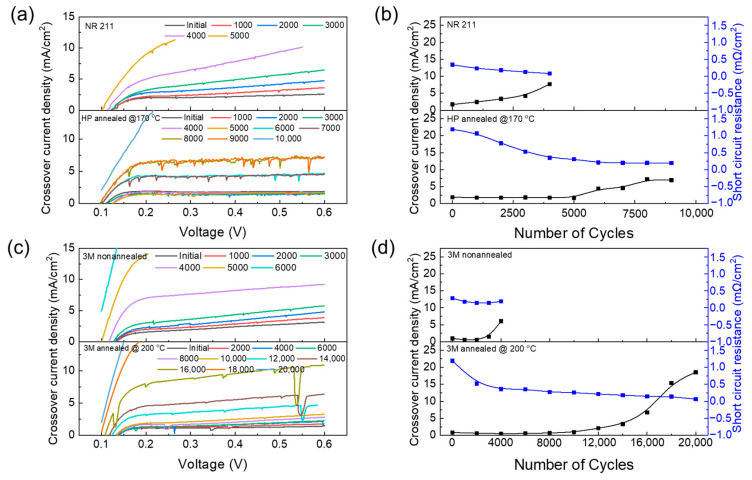
LSVs measurements and CCD and SCR of (**a**,**b**) NR 211, annealed Nafion HP, and (**c**,**d**) non-annealed and annealed 3M PFSA RCM.

Electrochemical assessments (LSV, CV, and EIS) were systematically conducted every 1000 cycles to facilitate periodic evaluations. Figure 3a,c illustrate the dynamics of the hydrogen crossover current density (CCD) of membranes and their short-circuit resistance (SCR) as a function of time. A distinct difference was noted in the CCD of the four membranes after 4000 cycles, where the CCD of NR 211 and 3M PFSA RCM increased at current densities greater than 10 mA cm^−2^. Annealed Nafion HP and 3M PFSA RCM failed after 9000 and 18,000 cycles, respectively. Slight variations were observed in the initial CCD values for different membrane types (Figure 3b,d). For non-annealed and annealed 3M PFSA RCMs, the CCD values were measured at 0.6 V as well as 0.99 and 0.80 mA cm^−2^, respectively, and for NR 211 and annealed Nafion HP, they were measured at 0.6 V as well as 1.72 and 1.88 mA cm^−2^, respectively.

Moreover, the CCD of NR 211 and non-annealed 3M PFSA RCM slightly decreased owing to tilting, rendering measurements after 4000 cycles unfeasible because of the considerable tilting of the LSV line. In contrast, the CCD of annealed Nafion HP and 3M PFSA RCM demonstrated durability, maintaining consistent values up to the first 5000 and 10,000 cycles, respectively. Notably, the CCD of annealed 3M PFSA RCM remained below ten times its initial value (criteria for test-stopping judgment) after 16,000 cycles, highlighting the considerable impact of annealing on membrane performance.

In addition to CCD analysis, SCR profiles further elucidated membrane deterioration. SCR indicates an undesirable condition where electrons bypass the intended electrochemical pathway, directly bridging the cathode and anode through the membrane rather than traversing the cell circuit. The initial SCR values were notably different for the four membranes; the SCR value of non-annealed 3M PFSA RCM (0.28 mΩ cm^−2^) was considerably lower than that of its annealed counterpart (1.22 mΩ cm^−2^). A similar pattern was observed in the comparison between NR 211 and annealed Nafion HP, where the initial SCR of NR 211 (0.34 mΩ cm^−2^) was notably lower than that of annealed Nafion HP (1.92 mΩ cm^−2^). A declining SCR reflects membrane degradation, especially its thinning due to COCV-AST, which leads to elevated CCD levels [25,26]. Moreover, the initial voltage drop observed across the first 1000 cycles was comparable across all tested membranes, suggesting similar starting conditions for all membranes at the onset of the experiment [27,28].

The annealing process was shown to be crucial in increasing membrane durability based on the lower CCD and higher SCR of annealed membranes compared with their non-annealed counterparts. Annealing helped improve the structural integrity of membranes, which reduced their susceptibility to degradation and thinning during COCV-AST. This ultimately contributed to an enhancement in their long-term performance and durability in fuel cell applications [10,29,30].

Using CV and focusing on the ECSA of various membranes, the degradation of the catalyst layer within MEAs during COCV-AST could be assessed. The initial ECSA values of NR 211 as well as annealed Nafion HP, non-annealed, and annealed 3M PFSA RCM membranes were similar (42.6, 52.3, 45.5, and 50.5 m^2^ g^−1^, respectively), indicating similar initial catalytic activity for all membranes. No significant reduction was observed in the ECSA within the first 1000 cycles, suggesting stable catalyst performance.

**Figure 4:**
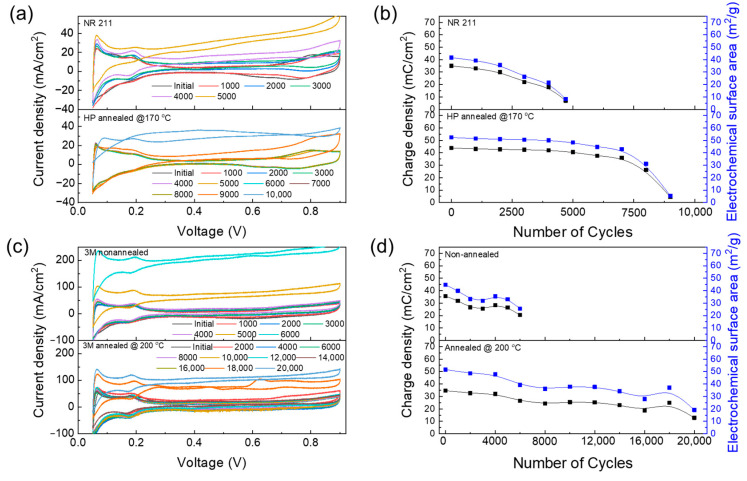
CVs and charge densities and electrochemical surface areas of (**a**,**b**) NR 211, Nafion HP annealed at 170 °C, and (**c**,**d**) non-annealed and annealed 3M PFSA RCM at 200 °C.

Because the catalyst layers used for preparing the four membranes were identical, the degradation of the PFSA membrane primarily resulted in ECSA changes [4,5]. In Figure 4a,c, NR 211 and non-annealed 3M PFSA RCM stabilized after 4000 cycles while annealed Nafion HP fully decomposed after 9000 cycles. In contrast, annealed 3M PFSA RCM demonstrated durability for up to 16,000 cycles. Figure 4b,d show the rapid reduction in the ECSA of NR211 and non-annealed 3M PFSA RCM, suggesting that the decomposition of the PFSA membrane increases gas permeability, which in turn boosts radical formation. These observations reveal that annealed PFSA membranes exhibit stable ECSA values, indicating that degradation is prevented in these membranes, which reduces their impact on the catalyst layer [4,5]. These results indicate that reducing the decomposition of the PFSA membrane enables the maintenance of low gas permeability and reduces the number of radicals generated in the electrode layer. Thus, the durability of ECSA values over time implies a direct correlation between reduced PFSA membrane decomposition and the performance durability of the catalyst layer [22,23,24]. This was confirmed by LSV observations, which considerably affected the CV patterns and highlighted the complex relationship among the PFSA membrane degradation, gas permeability, and durability of the MEA catalyst layer.

**Figure 5:**
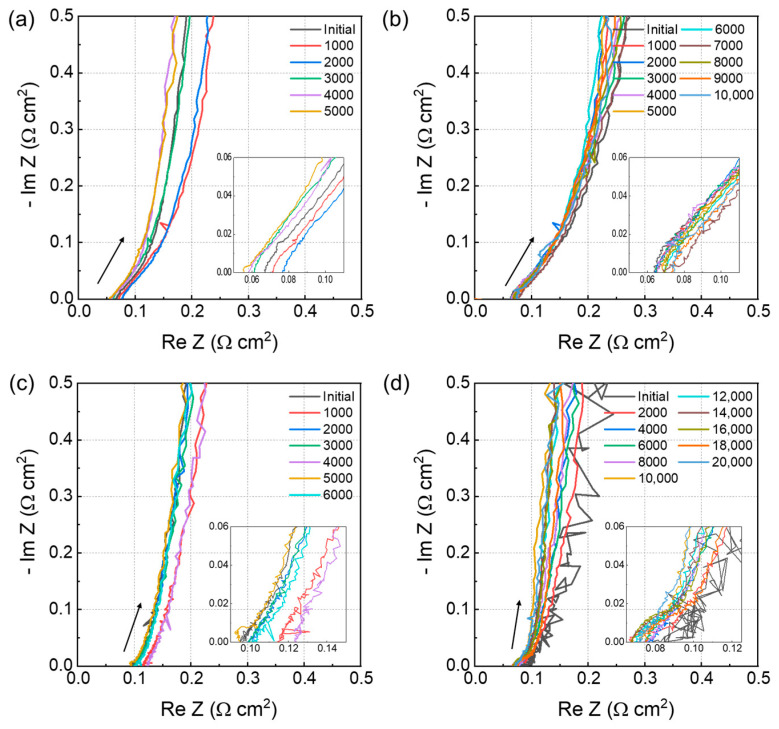
HFR curves of (**a**) NR 211, (**b**) annealed Nafion HP, and (**c**) non-annealed and (**d**) annealed 3M PFSA RCM (The arrows in each figure represents the slope of the curve).

**Figure 6:**
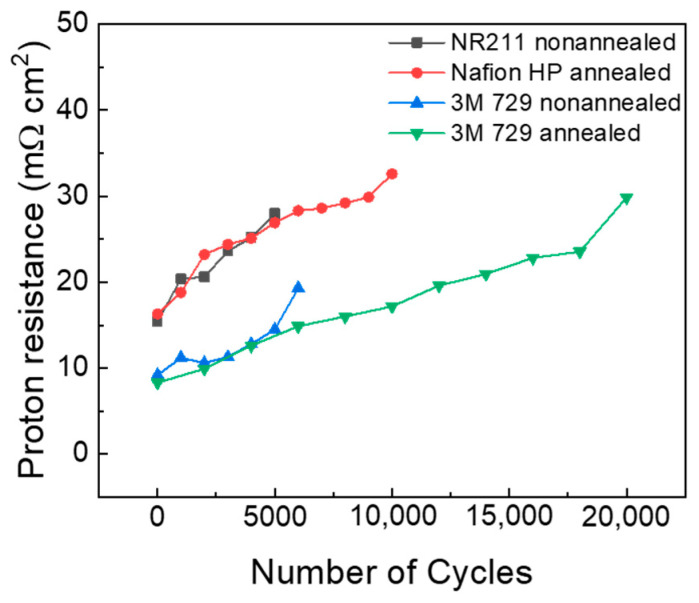
Proton resistances of NR 211 (black), annealed Nafion HP (red), non-annealed 3M PFSA RCM (blue), and annealed 3M PFSA RCM (green) MEAs over number of cycles.

The ionomer resistance of the electrode was monitored using EIS over the testing period, and the resistance was calculated using the transmission-line model [20]. The estimated value corresponds to the resistance of the ionomer in the catalyst layer. An increased proton resistance is attributed to the decomposition of the binder within the electrode due to the generation of radicals during the electrode operation. This degradation process affects the structural integrity and functionality of the ionomer, increasing proton conduction resistance within materials. Figure 5a–d show the monitoring of the EIS of all membranes over the test period, and Figure 6 illustrates the proton resistance of membranes throughout COCV-AST. The increase in proton resistance exhibits a close correlation with a decrease in the ECSA, indicating that the reduction in the ECSA is more likely a result of ionomer degradation rather than intrinsic changes in the catalyst itself [31].

The proton resistance of NR 211 increased from 15.4 to 28 Ω cm^2^ after 5000 cycles, and that of annealed Nafion HP increased from 16.3 to 32.6 Ω cm^2^ after 10,000 cycles. Moreover, those of non-annealed and annealed 3M PFSA RCM increased from 9.2 to 19.3 Ω cm^2^ after 6000 cycles and from 8.3 to 29.84 Ω cm^2^ after 18,000 cycles, respectively. The slope of the annealed Nafion HP curve was slightly less steep than that of non-annealed 3M PFSA RCM. Additionally, NR 211 exhibited the steepest slope, and annealed 3M PFSA RCM demonstrated the lowest slope among all membranes. With the degradation of the PFSA ionomer membrane, gas permeability increases, potentially increasing radical generation. Therefore, the increased proton resistance reflects the accelerated decomposition of the PFSA membrane and increased rate of radical generation. Radical generation will not increase if gas permeability does not increase, which will be reflected in the lower slope of the resistance curve.

These results indicate that the annealing process plays a crucial role in the relationship among gas permeability, radical generation, and proton resistance of membranes. By improving the structural integrity of membranes, annealing reduces their gas permeability, which in turn decreases radical generation. This explains the higher HFR and proton resistance of non-annealed membranes compared with annealed membranes, as shown in Figure 5c,d. Therefore, annealing helps maintain the durability and long-term performance of membranes by mitigating the accelerated decomposition and radical generation caused by increased gas permeability [5,10,22,29,30].

## 4. Conclusions

Herein, the complex relationship between the chemical and mechanical degradations and electrochemical performance of 3M PFSA RCMs, which are essential components of PEMFCs, was investigated. Using COCV-AST, our work explored the collective influence of the mechanical and chemical properties of membranes on fuel cell efficacy and longevity.

Our investigation revealed the exceptional resilience of annealed 3M PFSA RCMs under strenuous conditions, where annealing considerably enhanced their chemical and mechanical durability. The performance of annealed 3M PFSA RCMs surpassed that of the other investigated membranes because they endured up to 18,000 cycles before failure. In contrast, NR 211 lasted only for 5000 cycles, and the other tested membranes failed after 6000 (non-annealed 3M PFSA RCM) and 10,000 cycles (annealed Nafion HP). This superior performance of annealed 3M PFSA RCMs was attributed to the annealing process, which increased crystallinity and reduced free volume through recrystallization, strengthened membranes, and mitigated internal stress, microcrack, and pinhole formation induced by COCV-AST.

The findings of this study emphasize the importance of annealing for enhancing the mechanical and chemical robustness of PFSA RCMs, offering valuable insights into the design and engineering of more durable membranes. Furthermore, correlations among the ECSA, HFR, and proton resistance during COCV-AST enhance the understanding of the membrane behavior under stress. Our work provides a basis for future advancements in fuel cell technology for the development of PEMFCs with improved durability and operational efficiency.

## Data Availability

The original contributions presented in the study are included in the article, further inquiries can be directed to the corresponding author.
